# Impact of Land Use on Concentrations of Potentially Toxic Elements in Urban Soils of Lagos, Nigeria

**DOI:** 10.5696/2156-9614-8.19.180904

**Published:** 2018-08-21

**Authors:** Abimbola O Famuyiwa, Yetunde A Lanre-Iyanda, Olabode Osifeso

**Affiliations:** 1 Department of Science Laboratory Technology, Moshood Abiola Polytechnic, Ojere, P.M.B 2210 Abeokuta Ogun State, Nigeria

**Keywords:** soil, Lagos, heavy metals

## Abstract

**Background.:**

Among soil contaminants, potentially toxic elements (PTE) are of major significance because they are ubiquitous, toxic and persistent. Chronic exposure of humans to these elements has been linked with developmental delay, cancer, atherosclerosis and kidney damage, stomach ailments, respiratory problems, heart disease and cancer.

**Objectives.:**

The present study aims to investigate current PTE concentrations in urban soils of Lagos, an example of a rapidly urbanizing megacity in a developing country. The variation in PTE (chromium (Cr), copper (Cu), iron (Fe), magnesium (Mn), nickel (Ni), lead (Pb) and zinc (Zn)) levels across different land use types was examined. Information from this study will be useful in the ranking of contaminated sites, environmental quality management, guidance for remediation, redevelopment of contaminated sites and will provide crucial information for general urban planning decisions.

**Methods.:**

Five areas spread across four local government areas were selected, representing different socio-economic areas of Lagos (Victoria Island, Lagos mainland, Ikeja, Ifako-Ijaiye and Makoko). Sampling locations within the study areas were comprised of school playgrounds, roadsides, ornamental gardens, open spaces, train stations, industrial estates and dump sites. A total of 126 samples were collected.

**Results.:**

The overall mean levels of PTE concentrations in this study were comparable to those found in large European cities where main pollution sources include traffic and current or former heavy manufacturing industries.

**Conclusions.:**

Regulation and legislation on environmental issues, including effective solid waste management strategies and enforcement of emission standards should be emphasized in order to reduce the impact of PTE pollution on the inhabitants of urban areas in developing countries.

**Competing Interests.:**

The authors declare no competing financial interests

## Introduction

Soil functions as a pollution sink, but soil is also a source of pollution with the capacity to transfer pollutants to ground water, the food chain and eventually to the human body.^1^,^2^ Over the last few decades, high levels of urban soil pollution have become a major issue. Urbanization and industrialization have resulted in the release and discharge of pollutants and other persistent toxic substances, leading to the degradation of environmental conditions. Among soil contaminants, potentially toxic elements (PTE) are of major significance because they are ubiquitous, toxic and persistent. The prolonged presence of metals in urban soils and their close proximity to human populations can greatly amplify the exposure of these populations to metal contamination via inhalation, ingestion and dermal contact.^3–5^ Some of the elements found in soil are essential to living organisms, but can be toxic and dangerous to human health if present in soil above critical levels. The fate and transport of these elements in soils largely depend on the chemical form in which they exist. These elements associate with soil particles by different mechanisms and changes in conditions can bring about their release into the environment. The reaction mechanisms involve both physical and chemical processes including cation exchange, adsorption and desorption, co-precipitation, and organic complexation.^6^,^7^ Chronic exposure of humans to these elements has been linked with developmental retardation, cancer, atherosclerosis and kidney damage, stomach ailments, respiratory problems, heart disease and cancer. Urban soil quality has been investigated for many different parameters and in several different ways, including studies of PTE concentrations in roadside soils, parks, school playgrounds, sports grounds, different particle sizes of urban soil, comparative studies between rural and urban soils, the influence of large cities on PTE distribution, PTE concentration in urban soil at increasing depths, and effect of compost application on PTE load.^8–21^ These studies have demonstrated that trace metal contamination of the urban environment can have long term and far reaching environmental and health implications. Lagos, Nigeria has experienced an unprecedented sporadic increase in urban migration which may be due to the fact that it serves as the commercial capital of Nigeria.^22^ Over sixty percent of Nigeria's total industrial activities take place in Lagos.

In the long term, this results in environmental degradation and ultimately has the potential to endanger human health. The few studies available have demonstrated PTE contamination of Lagos soils, requiring further comprehensive investigation. However, there has been no general assessment of the PTE status of urban soils across Lagos. Previous studies have focused on PTE concentration measurements, but little effort has been devoted to determining sources and distribution across various land uses. Therefore, the present study aims to conduct a comprehensive investigation of current PTE concentrations in urban soils of Lagos, an example of a rapidly urbanizing megacity in a developing country. The variation in PTE (chromium (Cr), copper (Cu), iron (Fe), magnesium (Mn), nickel (Ni), lead (Pb) and zinc (Zn)) levels across different land use types was investigated. Information from this study will be useful in the ranking of contaminated sites, environmental quality management, guidance for remediation, redevelopment of contaminated sites and will provide crucial information for general urban planning decisions.

## Methods

Lagos is Nigeria's most populous city and the ninth fastest growing city in the world.^23^ A recent report estimated its population at 21 million, making Lagos the largest city in Africa.^24^ Lagos State is made up of 20 local government areas. For the purpose of this study, five areas spread across four local government areas were selected, representing different socio-economic areas of Lagos (Victoria Island, Lagos mainland, Ikeja, Ifako-Ijaiye and Makoko). The choice of sampling areas was based on income earnings (high, medium and low) and population densities specific to each of the areas. Victoria Island is the high income area, Lagos mainland, Ikeja and Ifako are medium income areas while Makoko is the low income area.

**Abbreviations**
*EF*: Enrichment factor
*Igeo*: Geoaccumulation index
*PTE*: Potentially toxic elements

**Figure 1:**
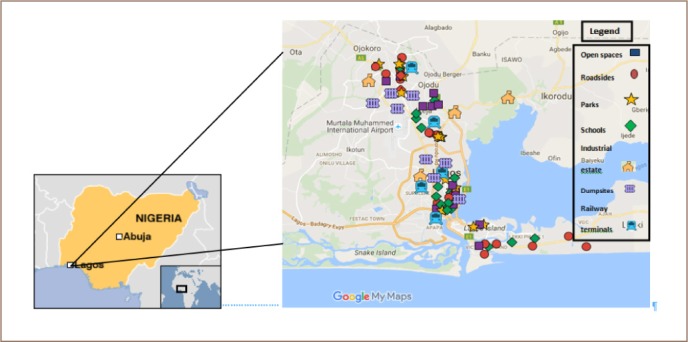
Lagos area map showing sampling points

Sampling locations within the study areas were comprised of school playgrounds, roadsides, ornamental gardens, open spaces, train stations, industrial estates and dump sites *(Figure 1)*. A total of 126 samples were collected in two months — February and August of 2014.

The sampling points in each of the locations were selected giving preference to places where people frequently relax or spend time. In the case of the school playgrounds, samples were collected at locations where children normally play. Soils were also collected from pedestrian walkways along the sampled roadsides. The industrial estates were co-located with residential layouts. The distance separating the two land-use types was about 20 to 30 m. Residential areas typically occupy about 30% of the total land space within the estates. Waste management of the estates is generally poor and most of the factories do not have installed emission control monitoring systems. Most of the sampled dumpsites are open dumps with heaps of waste littered around with no proper demarcation, less than 50 m from pathways alongside roads. Sampled railway terminals are major train terminals located in the metropolis and are accessible to users because of their closeness to residential settlements, workplaces, markets and schools.

At each sampling point, representative samples consisting of 5 – 10 sub-samples were obtained using a hand auger (at a depth of 0 – 10 cm). Polythene bags, nylon materials, grass and leaves were removed from the soil samples. Approximately 500 g of bulked sample was taken at each location. The coordinates of sampling locations were recorded using a Garmin GP Snap 60CSx. Soil samples were kept in sealed air tight polythene bags and labelled accordingly. Soil samples were then air dried in the laboratory for 20 days, ground and sieved through a 1 mm mesh. Samples were subjected to repeated reduction by coning and quartering processes prior to digestion.

### Sample digestion and analysis

Reagents used in this study were of analytical grade (Sigma Aldrich, Gillingham, UK) and a commercially prepared stock solution of multi-element standard supplied by Agilent UK was used to prepare the calibrants. Soil samples were subjected to closed microwave, *aqua regia* digestion to determine pseudototal PTE contents.^25^,^26^ Replicate samples (n = 3) were digested along with procedural blanks. Inductively coupled plasma mass spectrometry (ICP-MS model 7700× instrument, Agilent Technologies, UK) was used to determine PTE concentration in digests and extracts.

Instrument consistency was assessed intermittently by ensuring the calibration standards were checked after every tenth sample measured. A clear linear fit for the calibration curve for elements was obtained with regression coefficient (R^2^) of at least 0.999. The accuracy and precision of the analytical method employed was estimated by analyzing a certified reference material (BCR 143R; a sewage sludge amended soil) and the results were consistent with certified values.

### Assessment of heavy metal pollution

A common approach in assessing anthropogenic influence in soils is to calculate the enrichment factor (EF) for metal concentrations above background levels.^27^,^28^ Calculation of EF reduces PTE variability and is valuable in comparing the extent of pollution in absolute terms.^29^ The EF value is calculated by comparing measured PTE content with respect to a reference element.^30^, ^31^

The EF is calculated using Equation 1 below:

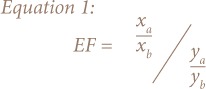
X_a_ is the concentration of PTE examined in the sample, x_b_ is the concentration of reference element in the sample, y_a_ is the background concentration of PTE examined and y_b_ is the background concentration of reference element.

Reference elements are normally chosen due to their lower variability and presence in trace amount in soils.^32^ In this study, Fe was chosen as the reference element. The geochemistry of Fe is similar to many PTEs and its standard deviation of successive measurements in the soil samples was generally good. Since background levels of PTE have not been established in Nigeria, the background concentrations of Cr, Cu, Fe, Mn, Ni, Pb and Zn published by Turekian and Wedepohl were used.^33^ The PTE values published by these authors were natural shale concentration values. Shale and world average soil or Earth crust values are often used to provide background reference values for PTE.^32^,^34^ Sutherland empirically suggested five categories of degree of pollution based on enrichment factor:^35^

EF < 2 No or minimal enrichment
EF = 2 - 5 Moderate enrichment
EF = 5 – 20 Significant enrichment
EF = 20 – 40 Very high enrichment
EF > 40 Extremely high enrichment

### Geoaccumulation index

The geoaccumulation index (Igeo) expresses the contamination levels of soils by comparing current PTE levels to those of non-anthropogenically influenced soils.^34^ The Igeo is used to assess the degree of contamination in soils and is expressed using Equation 2 below:

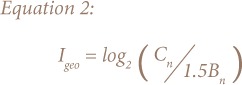
Where C_n_ isthe average concentration of PTE in the soil sample and B_n_ is the background geochemical Earth crust value. A factor of 1.5 was introduced in order to compensate for possible variations in background values which may be attributed to lithogenic effects. The Igeo is classified as follows:^30^

Igeo < 0 Practically unpolluted
Igeo 0–1 Unpolluted to moderately polluted
Igeo 1–2 Moderately polluted
Igeo 2–3 Moderately to strongly polluted
Igeo 3–3 Strongly polluted
Igeo 4–5 Strongly to extremely polluted
Igeo > 5 Extremely polluted

## Results

The quality of the pseudototal PTE analysis was assessed using BCR 143R, which is sewage sludge amended soil which was digested alongside the soil samples. The relative standard deviation for pseudo-total PTE analysis was generally less than 10% (n = 5) and average (%) PTE recoveries were adequate. Table 1 shows values in close agreement with the targets. Soil pH measured in the soil samples ranged between 5.79 and 11. The ornamental gardens soils were slightly alkaline with a mean pH value of 7.5. Overall mean concentrations of Cr, Cu, Fe, Mn, Ni, Pb, and Zn are presented in Table 2.

**Table 1:** Recovery of Certified Reference Material (BCR 143R)

**(n =5); mg/kg**	**Cr**	**Cu**	**Fe**	**Mn**	**Ni**	**Pb**	**Zn**
***Obtained values***	420	141	29700	871	297	173	1124
***Certified values***	426	—	—	858	296	174	1055
***Recovery %***	99			102	100	99	107
***SD***	31	8	1777	48	19	9	71
***% RSD***	8	5	6	5	6	5	6

Abbreviations: RSD, residual standard deviation; SD, standard deviation

**Table 2:** Average PTE Concentrations of Urban Soils (mg/kg) in Cities Around the World

City	Cr	Cu	Mn	Ni	Pb	Zn
***Lagos, Nigeria (this study)***	77	198	525	34	141	511
***Aberdeen, Scotland*^36^**	22	44	264	15	172	113
***Auby, France*^37^**	60.5	71.7		36	1118	1340
***Bangkok, China*^38^**	26	42		25	48	118
***Berlin, Germany*^39^**	18	19		5	41	101
***Damascus, Syria*^40^**	57	34		39	17	103
***Glasgow, UK*^41^**	45	85		35	307	199
***Gaborone, Botswana*^42^**	72	36		48	112	248
***Hong Kong, China*^43^**	18	16		4	88	103
***Ibadan, Nigeria*^44^**	64	47		20	95	228
***Oslo, Norway*^45^**	32.5	31.7	486	28.4	55.6	160
***Ottawa, Canada*^46^**	44.8	13.19		16.3	64.6	114
***Naples, Italy*^47^**		63			56	84
***Poznan, Poland*^48^**		10			17	32
***Rostock, Germany*^49^**	48	35		30	83	100
***Madrid, Spain*^11^**	75	72	477	14	161	210
***Seville, Spain*^50^**	39	68	471	22	137	145
***Tallinn, Estonia*^51^**	40	45		16	75	156
***UK Environmental Agency CLEA Guideline*^52^**	200			200	450	
***Dutch List Guideline*^53^**		190		210	530	750

Soils collected from open spaces, roadside and school playgrounds all had a mean pH value of 8. The average pH value for dumpsite soils was 7.6 followed by industrial estates soils (7.4) and train stations soils (7.1). The % loss on ignition values measured in the soils was generally low, ranging from 0.11 to 8.24. For all the samples analyzed, only three soils had organic matter above 5%. The low % loss on ignition values in these soils are typical of soils in warm arid climates like Nigeria.^30^

## Discussion

The average PTE values measured in soils were below the soil quality guideline values for the Netherlands and the United Kingdom, with exception of Cu.^54^ The overall mean PTE levels in Lagos urban soils (present study) were compared to a previous study on PTE levels in soils in Ibadan (capital of a neighboring state to Lagos).^44^ The results showed that PTE concentrations were much higher in Lagos soils than those in Ibadan soils. A study conducted by Iwegbue on the determination of PTE contents of soils collected from various land uses in Benin metropolis and Benin city (capital of a southern state in Nigeria) revealed lower PTE concentrations compared to Lagos urban soils, except for Pb.^55^ The overall mean levels of PTE concentrations in this study were comparable to those found in large European cities where main pollution sources include traffic and current or former heavy manufacturing industries. For instance, the levels of Cr, Cu, Mn, Ni and Pb in Lagos soils were higher than those found in cities such as Aberdeen, Scotland; Naples, Italy; Poznan, Poland; Tallinn, Estonia; Seville, Spain; Madrid, Spain and Rostock, Germany *(Table 2)*.^21^,^36^,^47^,^48^,^50^,^51^,^55^ The very high PTE concentrations measured in the industrial estates and dumpsites were responsible for the higher PTE concentrations.

However, the average Pb concentrations in Lagos soils were lower than those found in soils in Glasgow, UK and Auby, France.^37^,^41^ This may be attributed to the long histories of industrialization spanning several decades in these locations. It should be noted that much of the PTE pollution in industrial cities of Europe is historical rather than current, due to the decline in heavy industry and improvements in environmental protection. A previous study has attributed the degree of PTE pollution in urban areas to time variation.^56^ Comparisons between studies are somewhat relative and reported averages and ranges are all based on the methodology of a particular study. The non-uniformity of sampling strategies, study area, sampling locations (different land uses), number of samples, different sampling depths and extraction protocols can be a serious constraint in comparing published data. Because of the potential toxicity of PTE in urban soils and the threat to human health, there is a need for standardization of methodologies in studies of the geochemistry of urban soil.

**Figure 2:**
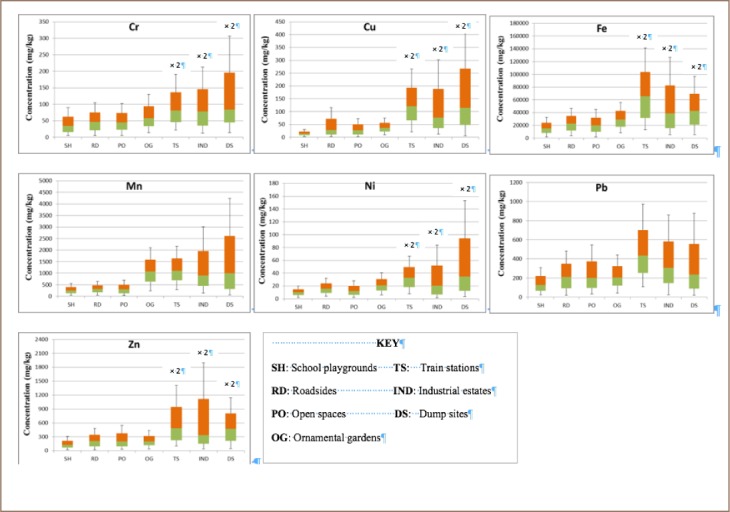
Box plots showing the variability of potentially toxic elements concentrations across different land use types. Chromium, Cu, Fe, Ni and Zn plots for train stations, industrial estates and dump sited are ½ of actual concentrations.

In terms of the influence of different land uses on PTE distribution *(Figure 2)*, as expected there was a clear distinction between the soil samples. The box plots showed Lagos soils varied widely in concentration, reflecting an array of lithogenic and anthropogenic impacts such as traffic emissions, industrial emissions, waste (domestic and electronic) disposal, soil transport and redistribution. The PTE concentrations were consistently higher in industrial estates, dump sites and train stations samples than in soils collected from school playgrounds, roadsides, open spaces and ornamental gardens.

For industrial estates, dump sites and train stations soils, Cr, Cu, Mn, Ni and Pb concentrations followed a consistent order of dump sites > industrial estates > train stations. This trend reflects a strong influence from industrial activities and deposition of domestic, electronic, scrap metal, and spent oil wastes in municipal dumpsites. Zinc had the highest concentration in industrial estates, followed by dump sites and train stations. Iron concentrations followed the order industrial estates > train stations > dump sites. Industrial estate soils were higher in Zn and Fe levels than dump sites soils. The concentrations of Zn and Fe measured in industrial estates soils were many times higher than the SGVs and typical soil concentrations.

Atmospheric emissions from smelter plants, steel, aluminum and galvanized pipe manufacturing at these sites may be responsible for the PTE enrichment in the soil samples. Indiscriminate dumping of scrap metals and improper disposal of wastes from these factories may have also contributed to the high levels of Fe and Zn in the soils. In comparison to school playgrounds samples, roadsides and ornamental gardens, open spaces samples presented the highest levels of Cu, Pb and Zn (37, 40 and 147 mg/kg, respectively). This may be because most of the open spaces sampled were bus stations. The contributions of direct emissions from vehicles and refuse burning to soils at these vicinities were apparent. Traffic-related emissions have been identified as one of the main sources of Pb pollution in urban soils.^9^,^57–62^ The lowest Pb concentrations were measured in soils collected from school playgrounds. This may be expected as soils collected away from traffic and known point sources are likely to be less subject to PTE contamination.^58^ Most of the school playgrounds soils sampled were located away from major roads and this may account for their lowest PTE concentrations in comparison to other land uses.

Levels of Cu, Pb and Zn were higher in ornamental gardens soils than school playgrounds samples. Reasons for higher PTE content may include enrichment from organic amendments and close proximity to traffic sources.^63^ Anthropogenic contributions to PTE levels are likely due to almost all of the sampled parks and gardens being located along major roads. Other studies have also reported PTE enrichment in urban park soils in relation to traffic sources.^50^,^57^,^64^ Another reason may be the possible addition of organic amendments to ornamental gardens soils, which may in turn have considerable PTE content, consequently increasing the PTE contents of soils.^63^ Concentrations of Cu, Pb and Zn followed a similar spatial distribution across the studied land use types (open spaces > roadsides > ornamental gardens > school playgrounds). This pattern indicates strong influence from vehicular emissions coupled with other anthropogenic PTE sources. Higher PTE concentrations were measured in open spaces and roadsides soils, which are more likely to be susceptible to vehicular pollution. Land use seems to similarly influence Cr, Fe, Mn and Ni, as their median and mean values followed the same pattern (ornamental gardens > open spaces > roadsides > school playgrounds). The highest concentrations for Cr, Fe, Mn and Ni were recorded in ornamental gardens soils, perhaps reflecting influence from lithogenic sources. This is likely, since the creation of new parks and green areas in Lagos began in 2008.^65^

Generally, Cr and Ni levels in ornamental gardens, open spaces, roadsides and school playgrounds showed low concentrations, supporting the earlier assertion that the primary source of PTE in this land use category likely has a lithogenic origin. In the present study, open spaces, roadsides, ornamental gardens and school playgrounds were similarly influenced by anthropogenic inputs (Cu, Pb and Zn) and this was evident in the decreasing concentrations of PTE measured in open spaces, roadsides, ornamental gardens (found along traffic corridors and in near proximity to industrial emissions) and school playgrounds (mostly located away from anthropogenic point sources).

**Figure 3:**
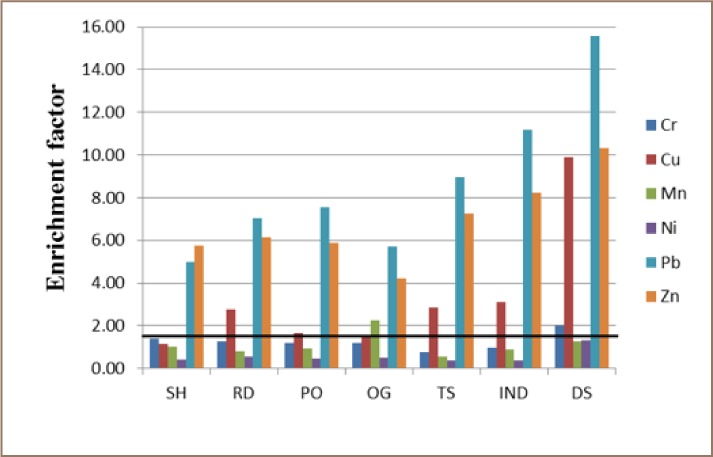
Enrichment factor showing the variability of potentially toxic elements contamination in different land use types. The bold horizontal line represents the limit for minimal enrichment.

**Figure 4:**
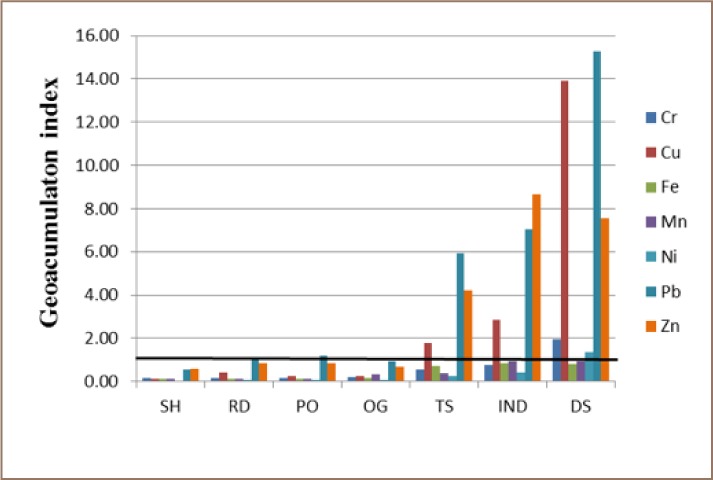
Geoaccumulation index showing the variability of potentially toxic elements contamination in different land use types. The bold horizontal line represents the limit of contamination.

Generally, the trend of Cr, Mn, and Ni pollution in all of the land uses followed a consistent order of dump sites > industrial estates > train stations > ornamental gardens > open spaces > roadsides > school playgrounds. For Cu and Pb, the PTE variability in the land uses followed the order of dump sites > industrial estates > train stations > open spaces > roadsides > ornamental gardens > school playgrounds. Zinc distribution across land uses followed the order of industrial estates > dump sites > train stations > open spaces > roadsides > ornamental gardens > school playgrounds and Fe distribution showed the order of industrial estates > train stations > dump sites > ornamental gardens > open spaces > roadsides > school playgrounds. It should be noted that school playgrounds consistently showed the lowest PTE concentrations for all of the land uses, and therefore was the least polluted, while dump sites was the most polluted. Enrichment factor and Igeo were computed to assess the possible anthropogenic contributions of PTE to studied soils. Figure 3 and 4 show the overall average EF and Igeo values, respectively. The average EF values for all land use types ranged from 0.08 – 6.0 (Cr), 0.58 – 74.1 (Cu), 0.48 – 6.4 (Mn), 0.26 - 4.4 (Ni), 1.0 – 61.7 (Pb), and 1.51 - 48.4 (Zn). It is evident from Figure 5 that Cr, Cu, Mn and Ni for school playgrounds, roadsides, open spaces and ornamental gardens soils were consistently below 1.5, indicating the soils in this land use category ranged between minimal and moderate enrichment. Similarly, minimal enrichment was observed Cr, Mn and Ni in industrial estates, dump sites and train stations soils. Only a few soil samples exceeded the minimal enrichment value of 1.5. This suggests that a large proportion of Cr, Mn and Ni in the soils may have a lithogenic rather than anthropogenic origin. Copper showed moderate to significant enrichment in roadsides, industrial estates, dump sites and train stations soils. It is apparent that Cu enrichment in these areas signifies anthropogenic contribution which may be from industrial emissions, car exhaust or electronic waste.

**Table 3:** Pseudototal PTE Concentration Across Land Use Types

	**Cr**	**Cu**	**Fe**	**Mn**	**Ni**	**Pb**	**Zn**
***SH***	20 ± 6	8 ± 2	7690 ± 374	132 ± 5	4 ± 2	17 ± 4	86 ± 32
***Range***	5 - 49	3 - 27	2230 - 19900	44 - 252	2 - 11	6 - 66	22 - 327
***RD***	23 ± 9	28 ± 9	9840 ± 3090	141 ± 42	7 ± 3	32 ± 18	121 ± 76
***Range***	5 - 38	3 - 124	3560 - 14200	39 - 213	4 - 19	5 - 66	19 - 403
***PO***	23 ± 11	37 ± 8	10600 ± 5360	170 ± 91	7 ± 4	40 ± 4	147 ± 15
***Range***	5 - 53	3 - 485	2210 - 27500	30 - 430	2 - 19	1 - 139	34 - 757
***OG***	28 ± 11	16 ± 4	11900 ± 3550	439 ± 96	9 ± 2	29 ± 12	97 ± 41
***Range***	14 - 51	10 - 22	8070 - 22200	230 - 557	6 - 16	18 - 57	42 - 198
***IND***	100 ± 8	194 ± 24	61000 ± 5500	1160 ± 141	42 ± 7	211 ± 16	1230 ± 145
***Range***	25 - 242	25 - 759	10900 - 146000	135 - 3950	4 - 118	23 - 504	87 - 3650
***DS***	262 ± 44	938 ± 288	55600 ± 4390	1200 ± 147	137 ± 25	515 ± 105	1070 ± 151
***Range***	19 - 1830	9 - 11700	10900 - 166000	54 - 6090	8 - 1040	22 - 4330	96 - 5600
***TS***	80 ± 37	190 ± 23	57500 ± 239	542 ± 310	28 ± 10	206 ± 90	614 ± 438
***Range***	43 - 128	43 - 710	26400 - 83600	280 - 1200	16 - 44	110 - 329	210 - 1220
***UK Environmental Agency CLEA Guideline*^52^**	200				200	450	
***Dutch List Guideline*^53^**	380	190			210	530	720

Abbreviations: DS, Dump sites; IND, industrial estates; OG, ornamental garden; PO, open spaces; RD, roadsides; SH, school playground; TS: Train stationsa ± represents standard deviation between successive measurements;

Lead and Zn showed moderate to significant enrichment in all of the land use areas. As expected, very high enrichment values for Pb and Zn were recorded for a considerable number of samples in industrial estates, dump sites and train stations soils. To a very large degree, Igeo results agreed with EF results. The same contamination pattern of Pb > Zn > Cu > Cr > Mn > Ni was observed for both EF and Igeo. The “typical urban metals” (Cu, Pb and Zn)^21^ had consistently higher enrichment factors and geo-accumulation index values for all studied land uses in comparison to other PTEs. The results of these pollution assessment tools strongly suggest that these PTEs were enriched in Lagos urban soils and Pb was the PTE of greatest concern.

Land uses appeared to have influenced PTE distribution in urban soils analyzed in the present study. The variability and lack of uniformity of PTE distribution in the studied areas can be attributed to different land use activities and disturbances, including duration and varied intensities of anthropogenic activities and geological characteristics of different soil types. This could alter the environmental stabilization of soils. The present study demonstrated PTE contamination of Lagos soils, particularly industrial estate and dumpsite soils. In developing countries such as Nigeria, the anthropogenic contribution of PTE to soils and associated health impacts may be the likely causes of increasing respiratory diseases, premature mortality and morbidity.^66^,^67^ Health impacts are more pronounced due to lack of environmental law enforcement, inadequate pollution management, emission standards, and poorly maintained vehicles.^68^,^69^ Economic development and industrialization, which in turn influence continued rural-urban migration, occurred prior to environmental protection in developing countries. Such a high rate of growth in cities in developing countries has implications for the provision of urban infrastructural services which could prevent a continued increase in informal urban settlements. This is evident in some of the studied areas where houses are built directly behind dumpsites and some industrial estates are co-located with residential settlements. In developed countries, the variability of PTE content across different land use types may exert less impact due to effective land use strategies, improved environmental control measures and a ban on leaded petrol. This conclusion is supported by the findings of Davidson et al., who found no relationship between PTE distribution and land use in five European cities.^70^ The results of the current study demonstrate that different land uses vary in PTE content and may exert different degrees of health impact on humans.

## Conclusions

The results of the present study demonstrated that urban soils in Lagos, Nigeria are characterized by low organic matter content and pH. Pseudo-total acid digestion of the soil samples demonstrated that PTE concentrations at locations remote from potential point sources of pollution were generally low, but soils taken from locations close to industrial activities and dumpsites were often highly contaminated. School playground soils consistently showed lower PTE concentrations for all the land use areas. There were indications that the higher PTE concentrations measured in open spaces and roadside soils might have been influenced by vehicular pollution when compared with low PTE contents recorded in school playgrounds soil. There was variability and lack of uniformity of PTE distribution in the studied land use types. Further studies of PTE mobility and speciation are recommended for the development of evidence-based testing and remediation. However, soil testing for elements that are above the threshold for soil guidelines should be done prior to building housing on sites where previous uses have demonstrated elevated pollutions levels in soil.

Regulation and legislation on environmental issues, including effective solid waste management strategies and enforcement of emission standards should be emphasized in order to reduce the impact of PTE pollution on the inhabitants of urban areas in developing countries.
